# Powassan Virus Lineage I in Field-Collected *Dermacentor variabilis* Ticks, New York, USA

**DOI:** 10.3201/eid2902.220410

**Published:** 2023-02

**Authors:** Charles Hart, Erin Hassett, Chantal B.F. Vogels, Daniel Shapley, Nathan D. Grubaugh, Saravanan Thangamani

**Affiliations:** Upstate Medical University, Syracuse, New York, USA (C. Hart, E. Hassett, S. Thangamani);; Yale School of Public Health, New Haven, Connecticut, USA (C.B.F. Vogels, N.D. Grubaugh);; Upstate Tick Testing Program, Syracuse (D. Shapley);; Yale University, New Haven (N.D. Grubaugh)

**Keywords:** Powassan virus, *Dermacentor variabilis*, dog tick, vector-borne infections, New York, ticks, United States, viruses

## Abstract

Powassan virus is a tickborne flavivirus that can cause lethal or debilitating neurologic illness. It is canonically transmitted by *Ixodes* spp. ticks but might spill over to sympatric *Dermacentor* species. We detected Powassan virus lineage I from a pool of field-collected *D. variabilis* ticks in New York, USA.

Powassan virus (POWV) is a neurotropic, tickborne flavivirus first identified as a human pathogen in 1958, when it was isolated from the brain of a patient who had died of encephalitis (*1*). POWV infection results in febrile illness that can progress to encephalitis, meningitis, and, rarely, meningoencephalitis (*2*), which is associated with head pain, confusion, paralysis, coma, and death in up to 15% of cases. In addition, >50% of survivors experience long-term neurosequelae, including motor deficiency and cognitive deficits (*3*).

POWV was initially associated with the woodchuck tick, *Ixodes cookei* (*4*), although a second lineage was discovered in deer ticks (*I. scapularis*) (*5*). That genotype was termed POWV lineage II, or deer tick virus (DTV). Because of the frequency with which *I. scapularis* tick bites occur in humans compared to *I. cookei* tick bites, DTV is likely the most common etiologic source of Powassan encephalitis in the United States. However, the source is difficult to discern because of serologic homology between the virus lineages and the lack of viral genotyping in most clinical settings.

Recently, interest has grown in the vector competency of other sympatric tick species. *Dermacentor* spp. ticks have been of particular interest because of their common occurrence in POWV- and DTV-endemic areas and because of their tendency to bite humans. POWV has been isolated from *D. andersoni* ticks in Colorado, USA (*6*); genetic analysis suggests that strain, called POWV 791A-52, is most likely a form of DTV (*7*). However, it remains unclear whether the tick in question was infected by spillover from another sylvatic cycle featuring *Ixodes* ticks or constitutes its own sylvatic system. Neither *I. scapularis* nor *I. cookei* ticks are native to Colorado, although several rodent-specific *Ixodes* species are present (*8*) that might be involved in such a system.

The competency of *D. andersoni* ticks for POWV has been confirmed under laboratory conditions when fed from artificially inoculated nonnative species (*9*). Recent analysis has also indicated that *D. variabilis* ticks are capable of acquiring and transmitting DTV under laboratory conditions, including maintaining replicating virus transstadially (*10*). Although that capability has been confirmed in experimentally infected ticks, it remains unclear whether wild populations of *D. variabilis* ticks can maintain and transmit POWV or DTV under natural circumstances. Considering that *D. variabilis* ticks are the second most common human-biting species in New York, USA, (*11*), the ability for the species to transmit POWV in nature represents a critical component of potential human exposure. We detected POWV lineage I from *D. variabilis* ticks collected in New York in 2021.

## The Study

As a part of ongoing efforts to track the emergence of POWV in New York, we performed tick surveillance in areas known to contain circulating POWV as identified from a community-engaged tick testing program (*11*). From 1 area of interest in Dutchess County, New York, we collected 5 female and 3 male *D. variabilis* ticks, in addition to 68 *I. scapularis* ticks, in the second half of April 2021. We visually speciated the ticks and assessed them for feeding status. The female *D. variabilis* ticks were unfed; we pooled, homogenized, and tested them for POWV by quantitative reverse transcription PCR as described (*11*). In brief, we initially detected POWV with a primer sensitive to both POWV lineage I and DTV. Then, we used a differentiation quantitative reverse transcription PCR to confirm POWV lineage I with a titer of 3.88 log_10_ FFU/μg RNA. In contrast, none of the *I. scapularis* ticks collected from the same site tested positive for POWV lineage I and DTV.

We used our highly multiplexed PCR amplicon approach to sequence POWV detected from the tick homogenate (*12*). We prepared libraries with the Illumina COVIDSeq Test (RUO version; Illumina, https://www.illumina.com), replacing the SARS-CoV-2 primers with POWV (*13*), and sequenced on the Illumina NovaSeq at the Yale Center for Genome Analysis (New Haven, CT, USA). Consensus genomes were generated at a minimum nucleotide frequency threshold of 0.75 and minimum depth of 10 reads using iVar version 1.3.1 (https://github.com/andersen-lab/ivar). 

We reconstructed a maximum-likelihood phylogenetic tree of 29 aligned POWV genomes trimmed to the coding sequence (genome positions 108–10,352) (Figure) using IQ-TREE version 1.6.12 (http://www.iqtree.org) with ultrafast bootstrap approximation (1,000 replicates) (*14*). Our phylogenetic analysis revealed that the virus (deposited in GenBank under accession no. OM681505) belongs to the POWV lineage I clade and is closely related to a POWV lineage I virus that we sequenced from *I. cookei* ticks (GenBank accession no. OM681504) from New York in 2020 (Figure). In addition, we used Sanger sequencing of tick ribosomal RNA to confirm that the sample was derived from *D. variabilis* ticks and not another potential vector species. The resulting sequence (GenBank accession no. ON922563) had 100% homology to *D. variabilis* large subunit ribosomal RNA (GenBank accession no. L34300.1). 

**Figure:**
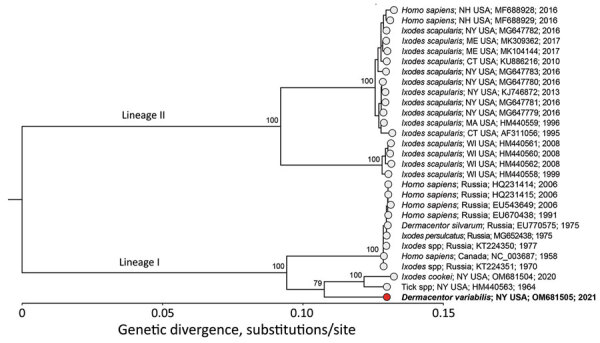
Maximum-likelihood phylogenetic tree of Powassan virus lineage I and II from *Dermacentor variabilis* ticks collected in New York, USA, and reference sequences. Phylogenetic analysis of the coding sequence (genome positions 108–10,352) of 29 publicly available Powassan lineage I and II genomes. Red circle and bold text indicate the virus sequenced in this study from the *D. variabilis* ticks; the virus is most closely related to other lineage I sequences from ticks in New York. Sequence names consist of host; location; GenBank accession number; year.

Our results confirm POWV in *D. variabilis* ticks in southern New York, suggesting that POWV can exist in this tick species, either because of incidental exposure or because of its own sylvatic cycle. In this study, the POWV we identified groups with lineage I; this lineage is normally associated with *I. cookei* ticks and woodchucks (*Marmota monax*), instead of with *I. scapularis* ticks and *Peromyscus leucopus* mice (*15*). This link suggests either spillover from that sylvatic cycle, a unique *D. variabilis* species-dependent sylvatic cycle for POWV lineage I, or a unique subtype of the virus specific to *D. variabilis* ticks with an unknown sylvatic cycle. Regardless of its source, our data indicate that some *D. variabilis* ticks in POWV-endemic areas could be capable of acquiring a genotype of POWV similar to lineage I.

## Conclusions

POWV is a medically noteworthy flavivirus understood to be primarily transmitted by *Ixodes* spp. ticks in North America. The sympatric tick species *D. variabilis*, however, has been recently demonstrated to be a competent vector for POWV under laboratory conditions (*10*). We report the detection of POWV lineage I in *D. variabilis* ticks collected from the wild, suggesting that the species might play a direct role in POWV transmission in nature. The ability of POWV to infect humans and the nature of the disease it causes remain unclear, and further research is needed to understand the role of *D. variabilis* ticks in the ecology of POWV. However, considering that *D. variabilis* ticks are a primary species of human-biting ticks in New York, this finding demonstrates a new potential source of human exposure to POWV. 
